# Editorial: Cell death in kidney diseases: novel biomarkers, mechanisms, and therapeutic strategies

**DOI:** 10.3389/fphar.2025.1715728

**Published:** 2025-12-12

**Authors:** Min Chen, Yu Ning Liu, Wei Jing Liu

**Affiliations:** 1 Key Laboratory of Chinese Internal Medicine of Ministry of Education and Beijing, Dongzhimen Hospital Affiliated to Beijing University of Chinese Medicine, Beijing, China; 2 Peking University Institute of Nephrology, Key Laboratory of Renal Disease, Ministry of Health of China, Beijing, China; 3 Dongzhimen Hospital, Beijing University of Chinese Medicine, Beijing, China

**Keywords:** cell death, kidney diseases, biomarkers, ferroptosis, therapeutics

Cell death is a significant factor in the pathogenesis of kidney diseases. Acute kidney injury can be triggered by the acute loss of renal epithelial cells. In chronic kidney disease, the loss of renal epithelial cells leads to glomerulosclerosis and tubular atrophy. The cell death mechanism, which is supposed to clear abnormal cells, can instead accelerate renal cell injury when imbalanced. Programmed cell death, including apoptosis and necrosis, has been confirmed as a central link in the pathogenesis of various kidney diseases and holds potential for therapeutic intervention. Regulated necrosis modes, such as ferroptosis, necroptosis, and pyroptosis, may directly cause renal injury or indirectly induce damage by recruiting immune cells and stimulating inflammatory responses (Sanz et al., 2023). Cell death modes have become innovative therapeutic targets for kidney diseases, and the modulation of various interconnected pathways of cell death also presents both challenges and opportunities for further exploration into the pathogenesis and pharmaceutical intervention of renal diseases.

Regarding the mechanisms of renal cell death, articles in this Research Topic summarize evidence for the specific activation of non-canonical cell death mechanisms (e.g., ferroptosis) in nephropathy. Ferroptosis is a novel form of cell death, regulated by the lipid repair enzyme glutathione peroxidase 4 (GPX4) and induced by pronounced lipid peroxidation, dependent on reactive oxygen species (ROS) generation and excess iron. The process of ferroptosis is often accompanied by substantial iron accumulation and lipid peroxidation (Li et al., 2020). Huang et al. investigated the mechanism by which endoplasmic reticulum stress (ERS) controls ferroptosis, explored the relationship between ERS and heat shock protein family A member 5(HSPA5), and proposed a novel theory that ERS influences ferroptosis in tubular epithelial cells (TECs) during epithelial-mesenchymal transition (EMT) in renal fibrosis, providing a theoretical basis for the treatment of patients with chronic kidney disease (CKD). Hu et al., through a bibliometric analysis of literature from 2012 to 2024, provided comprehensive information on the publication landscape concerning ferroptosis in kidney diseases and noted that a growing number of studies suggest the potential of targeting ferroptosis, particularly through the accumulation of lipid peroxides, for treating kidney diseases. Concurrently, articles in this Research Topic have also explored how multiple cellular pathways, beyond cell death, influence various kidney diseases. Liang et al. found that in IgAN renal tissues and Gd-IgA1-induced HBaN-1 cells, Luteolin (Lut) increased the expression levels of Nrf2, HO-1, and NQO1, suggesting the involvement of the Nrf2/HO-1 pathway in protecting the kidney from Gd-IgA1-induced injury. Inhibition of Nrf2 suppressed the activation of Nrf2, HO-1, and NQO1 in the presence of Lut, leading to a reversal in the expression of extracellular matrix (ECM)-related proteins. Yan et al. demonstrated that Shenqi Yishen Formula (SQYSF) plays a role in ameliorating renal cell senescence in diabetic kidney disease (DKD) by targeting and reducing YTH N6-methyladenosine RNA Binding Protein F1 (YTHDF1) expression levels, thereby inhibiting Rubicon mRNA and protein translation levels and promoting autophagy. Therefore, Yan et al. reveal the active components and mechanism of SQYSF in treating DKD, potentially providing useful information for guiding the clinical application of SQYSF and therapeutic approaches for DKD.

In the area of biomarker research, articles in this Research Topic have explored the diagnostic potential of exosomes for kidney diseases. Cao et al. reviewed the roles of exosomes derived from different cell types within the kidney and discussed their physiological and pathological functions. Exosomes are nanoscale vesicles, 40–100 nm in diameter, released by various cell types into the extracellular space. They can transport a variety of bioactive substances which can influence various pathological processes associated with kidney diseases, exhibiting either detrimental or beneficial effects. Within the kidney, exosomes originating from glomeruli and tubules have the capacity to enter the systemic circulation or urine. The biomarkers they carry can reflect changes in renal pathological status, thereby offering new avenues for early diagnosis. However, exosomes also present certain limitations, such as difficulties in meeting the needs for rapid clinical detection, and the relatively small sample sizes used in studies of exosomal biomarkers in kidney diseases, necessitating further validation with larger clinical cohorts.

Regarding the mechanisms of action of pharmacological therapies, Qiu et al. employed network pharmacology, molecular docking, and *in vitro* experiments to investigate the mechanism of action of *Choerospondias axillaris* (Roxb.) Burtt et Hill (commonly known as the South Asian hog plum or “nansuanzao” in Chinese, a fruit-bearing tree valued for both its medicinal and edible uses, abbreviated as CA) in treating kidney stones. Their research indicates that CA can enhance cellular antioxidant capacity through multiple pathways, regulate NLRP3 inflammasome activation, and reduce the expression of the crystal adhesion protein osteopontin (OPN), thereby alleviating oxidative stress injury, inflammatory responses, and cell damage and adhesion induced by calcium oxalate monohydrate (COM) crystals. This helps prevent further aggregation or mineralization of calcium oxalate crystals, achieving the goal of treating kidney stones. Wen et al. utilized proteomics and network pharmacology to explore the relevant mechanisms of Huangkui Capsule (HKC) in treating chronic glomerulonephritis. Their study found that HKC alleviates oxidative stress, inflammation, and renal injury by modulating the STAT3/PIK3R1/AKT1/HIF-1α/VEGF signaling pathway, thereby ameliorating chronic nephritis.

The articles in this Research Topic demonstrate promising prospects in the research of cell death in kidney diseases from the aforementioned three aspects. The Editors anticipate that the articles published in this Research Topic will be of interest to readers and hope that researchers will benefit from them.

## References

[B1] Li J. Cao F. Yin H. L. Huang Z. J. Lin Z. T. Mao N. (2020). Ferroptosis: past, present and future. Cell Death Dis. 11 (2), 88. 10.1038/s41419-020-2298-2 32015325 PMC6997353

[B2] Sanz A. B. Sanchez-Niño M. D. Ramos A. M. Ortiz A. (2023). Regulated cell death pathways in kidney disease. Nat. Rev. Nephrol. 19 (5), 281–299. 10.1038/s41581-023-00694-0 36959481 PMC10035496

